# Maintenance of the critical care system during the pandemic in non-COVID-19 patients requiring continuous renal replacement therapy: a single center experience

**DOI:** 10.1186/s12873-022-00693-7

**Published:** 2022-08-01

**Authors:** Harin Rhee, Gum Sook Jang, Sungmi Kim, Wanhee Lee, Hakeong Jeon, Da Woon Kim, Byung-min Ye, Hyo Jin Kim, Min Jeong Kim, Seo Rin Kim, Il Young Kim, Sang Heon Song, Eun Young Seong, Dong Won Lee, Soo Bong Lee

**Affiliations:** 1 Department of Internal Medicine, Pusan National University School of Medicine, Yangsan, Republic of Korea; 2 Divison of Nephrology, Biomedical Research Institute, Pusan National University Hospital, 305 Gudeok-ro, Seo-gu, Busan, 602-739 South Korea; 3 Department of Nursing, Pusan National University Hospital, Busan, Republic of Korea; 4 Research Institute for Convergence of Biomedical Science and Technology, Pusan National University Yangsan Hospital, Yangsan, Republic of Korea

**Keywords:** COVID-19 pandemic, Critical care, Health care system, Continuous renal replacement therapy, Essential healthcare system, Triage system

## Abstract

**Background:**

During the COVID-19 pandemic, maintenance of essential healthcare systems became very challenging. We describe the triage system of our institute, and assess the quality of care provided to critically ill non-COVID-19 patients requiring continuous renal replacement therapy (CRRT) during the pandemic.

**Methods:**

We introduced an emergency triage pathway early in the pandemic. We retrospectively reviewed the medical records of patients who received CRRT in our hospital from January 2016 to March 2021. We excluded end-stage kidney disease patients on maintenance dialysis. Patients were stratified as medical and surgical patients. The time from hospital arrival to intensive care unit (ICU) admission, the time from hospital arrival to intervention/operation, and the in-hospital mortality rate were compared before (January 2016 to December 2019) and during (January 2021 to March 2021) the pandemic.

**Results:**

The mean number of critically ill patients who received CRRT annually in the surgical department significantly decreased during the pandemic in (2016–2019: 76.5 ± 3.1; 2020: 56; *p* < 0.010). Age, sex, and the severity of disease at admission did not change, whereas the proportions of medical patients with diabetes (before: 44.4%; after: 56.5; *p* < 0.005) and cancer (before: 19.4%; after: 32.3%; *p* < 0.001) increased during the pandemic. The time from hospital arrival to ICU admission and the time from hospital arrival to intervention/operation did not change. During the pandemic, 59.6% of surgical patients received interventions/operations within 6 hours of hospital arrival. In Cox’s proportional hazard modeling, the hazard ratio associated with the pandemic was 1.002 (0.778–1.292) for medical patients and 1.178 (0.783–1.772) for surgical patients.

**Conclusion:**

Our triage system maintained the care required by critically ill non-COVID-19 patients undergoing CRRT at our institution.

**Supplementary Information:**

The online version contains supplementary material available at 10.1186/s12873-022-00693-7.

## Background

Since declaration of the COVID-19 pandemic by the World Health Organization (WHO) on March 11th, 2020, there have been 149,359,111 reported cases and 3,149,381 reported deaths globally by April 28th, 2021 [1]. In South Korea, the first case of COVID-19 was reported on January 20, and up to April 28th, 2021, there have been 12,673 reported cases and 1821 (1.5%) reported deaths [2].

The Korean government actively responded to the pandemic, focusing on the treatment of COVID-19-infected patients by reorganizing existing health care resources [3]. They dedicated 67 hospitals to infectious diseases until March 2020 and further designated 2468 hospitals nationwide to have adequate health facilities dedicated to COVID-19 treatment by November 2020 [3, 4].

Reorganizing existing health care resources to cope with the COVID-19 pandemic may negatively affect essential health care services for non-COVID 19 patients due to the unprepared/underprepared medical health infrastructure, inadequate health workforce, shortage of personal protective equipment for health care workers, and lack of ventilators for critical care [5]. During the Ebola outbreak in West Africa in 2014–15, increased morbidity and mortality in other diseases were seen due to the reduction in access to and utilization of healthcare services, and death from these diseases outnumbered deaths from Ebola [6].

The critical care system is one of the most important components of essential health care services, and maintenance of this system in the absence of nosocomial infection has been an important challenge during the pandemic. Nevertheless, nation-level or institution-level outcomes on this issue have rarely been reported.

In this report, we describe the institution-level strategies that we used to manage critically ill patients visiting the emergency room and their outcomes during the pandemic.

## Methods

### Patient population

We retrospectively reviewed the medical charts of patients admitted to the intensive care unit (ICU) and receiving continuous renal replacement therapy (CRRT) from January 2016 to March 2021 without SARS-CoV-2 infections. We excluded end stage kidney disease (ESKD) patients who were on maintenance hemodialysis prior to admission, as well as patients admitted to the emergency department. We classified patients into two groups according to time period: Before (January 1, 2016 to December 31, 2019) and during the pandemic (January 1, 2021 to March 31, 2021). We stratified patients by major departments during hospital admissions. The surgical department included general surgery, trauma surgery, thoracic surgery, neurosurgery, and urology. The medical department included internal medicine, neurology, and rehabilitation medicine.

### The triage system used to manage critically ill patients visiting the emergency room during the COVID-19 pandemic

**Fig. 1:**
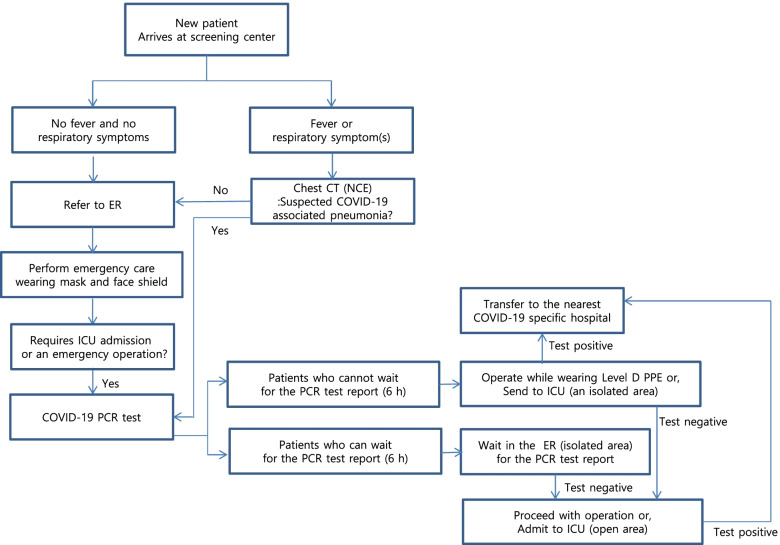
Flow chart showing our management of new, critically ill patients during the COVID-19 pandemic: ER, emergency room; ICU, intensive care unit; CT, computed tomography; PPE, personal protection equipment

Figure 1 shows the strategies that we employed during the COVID-19 pandemic. We focused on both timely provision of emergency interventions and prevention of nosocomial infection during both intensive care unit (ICU) stays and emergent surgery/interventions. Briefly, a screening center that preceded the emergency room triaged all patients in the contexts of respiratory symptoms or fever. If a patient lacked any clinical evidence of infection, we referred that patient to the emergency room. If an infection was evident, we performed computed tomography (CT) to exclude pneumonia. If CT revealed evidence of viral pneumonia, we performed a COVID-19 PCR test [7]. That test requires 6 hours. If the patient was unstable, and thus required an emergency intervention (bleeding control or a coronary procedure), that was provided; all medical staff wore Level D personal protection equipment (PPE) [8] regarding them as positive until a negative PCR test is obtained. Regardless of the CT findings, all patients requiring ICU admission or an emergency operation underwent a COVID-19 PCR test. This strategy was applied in all ICUs (Supplemental Table 1) and all emergency operation rooms of our institute.

### Variables of interest

We collected demographic data including age, sex, height, and body weight at ICU admission. Information regarding comorbidities, including diabetes, hypertension, cardiovascular disease, chronic kidney disease, liver disease, lung disease, and cancer were obtained based on the admission notes. The first hospital visit time and route of hospital admission (emergency department (ED), transfers from another hospital, and outpatient department (OPD)) were reviewed. For patients admitted to the surgical department, the time from hospital arrival to intervention/operation was calculated. Typical procedures performed in emergency care, such as vascular access, intubation, and temporary dialysis catheter insertion, were excluded from the intervention. Disease severity was assessed based on the organ failure status. We defined circulatory failure as vasopressor or inotropic medication requirement, respiratory failure as ventilator requirement, and kidney failure as dialysis requirement. Organ failure was assessed at the time of hospital arrival (within 24 hours) and at the time of CRRT initiation. Laboratory parameters were determined at the time of CRRT initiation. We assessed ICU and in-hospital mortality.

### Statistical analysis

Continuous variables are presented as the mean ± SD or median (interquartile range: IQR). Data normality was verified using the Kolmogorov-Smirnov test. Differences in the annual number of patients requiring CRRT before and during the pandemic were compared using a one-sample t-test. Differences in the continuous variables were compared using the Student’s t-test or the Kruskal-Wallis test, as appropriate. The categorical variables are summarized as numbers and percentages and compared using a chi-squared test. Univariable and multivariable analyses were performed via a Cox proportional hazard model. All statistical tests were two-sided, and we considered *p* < 0.05 to be significant. We analyzed the data with IBM SPSS (v.26.0, SPSS Inc., Chicago, IL, USA).

## Results

### Changes in the numbers, and the processes of ICU admission in critically ill patients requiring CRRT during the pandemic

**Fig. 2:**
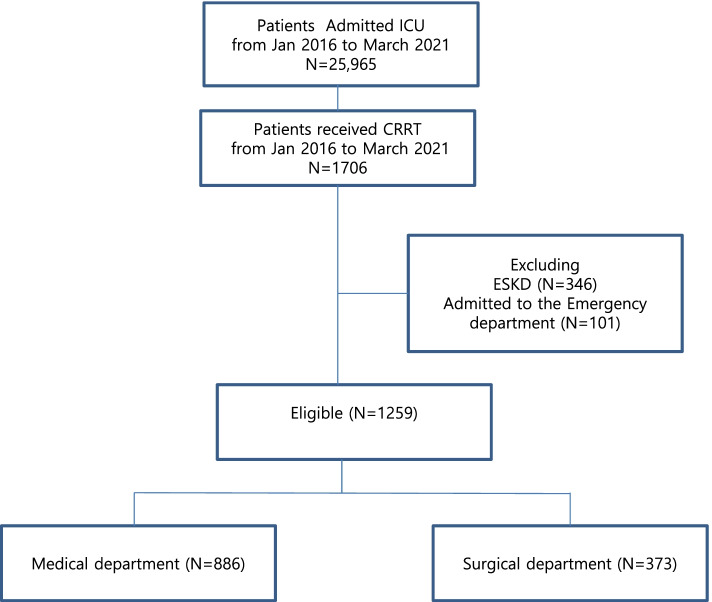
A consort diagram of the included patients

From January 2016 to March 2021, a total of 25,965 patients were admitted to the ICU and 1706 (6.6%) received CRRT at our hospital. By excluding 346 patients with ESKD who were on maintenance dialysis and 101 patients who admitted to the emergency department, 1259 patients were included in this study (Fig. 2). Among them, 886 (70.4%) were admitted to the medical department and 373 (29.6%) were admitted to the surgical department.

**Fig. 3:**
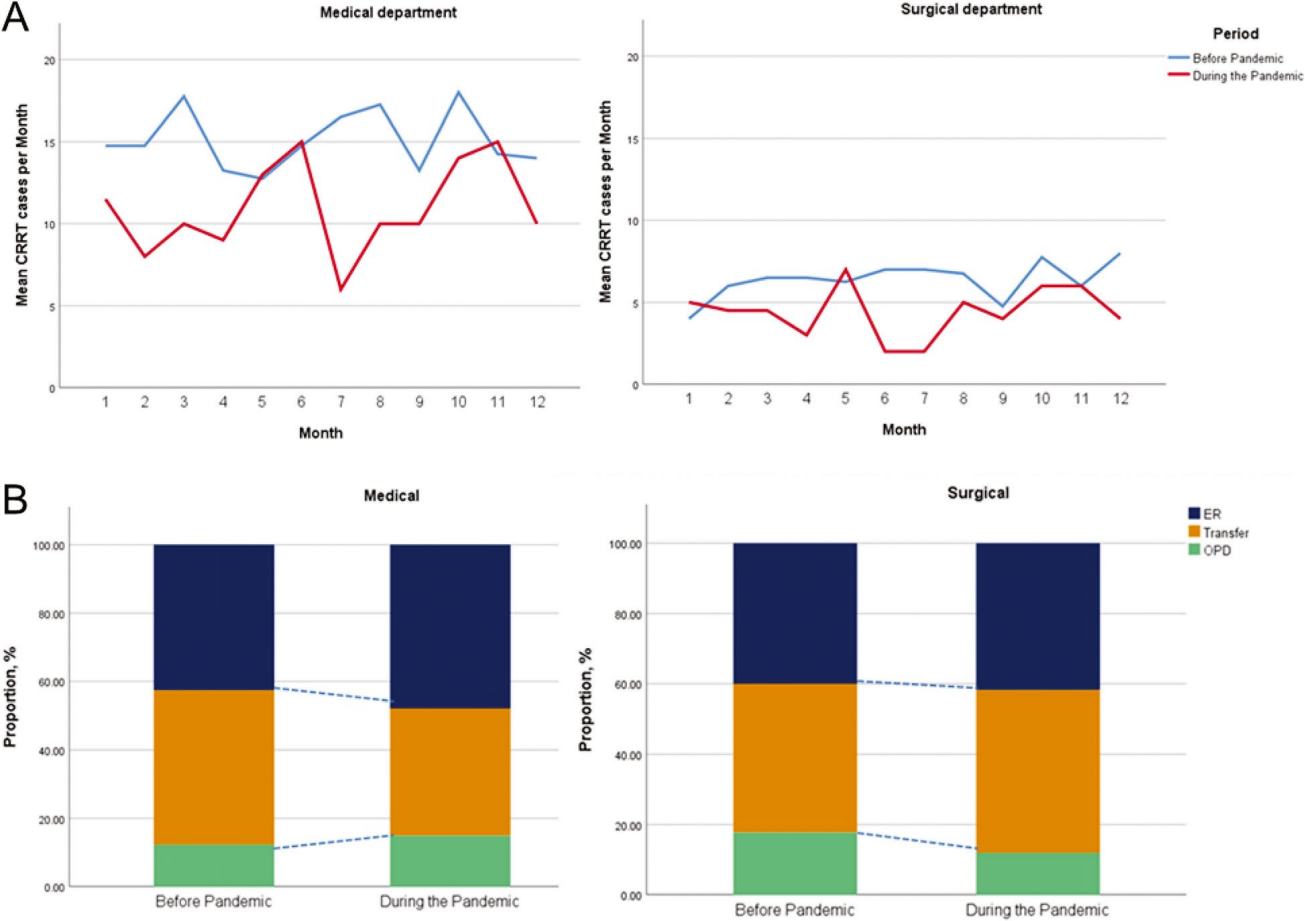
**A** The average monthly numbers of patients receiving continuous renal replacement therapy before and during the pandemic, stratified by the department of care. Blue line: before the pandemic; red line: during the pandemic. **B** The intensive care unit admission routes before and during the pandemic, stratified by the department of care. Navy line: admitted via the emergency department; orange line: transferred from other hospitals; green line: admitted via our outpatient department

Between Jan 2020 and Dec 2020, the total number of patients admitted to the ICU was not different from the annual number of ICU patients between Jan 2016 and Dec 2019 (Supplemental Table 2A). However, a significant decrease was seen in both the number (2020: 56, 2016–2019: 76.5 ± 3.1, *p* = 0.010) and proportion (2020: 2.0%, 2016–2019:2.7 ± 0.2%, *p* = 0.022) of CRRT-receiving patients in the surgical department, whereas no change was seen in the medical department (Supplemental Table 2B). Monthly changes in the number of patients receiving CRRT by period stratified by department are plotted in Fig. 3A.

Changes in the route of ICU access were evaluated in 1258 out of 1259 patients. In medical patients, 386 (43.6%) of 886 patients were admitted via the ED, 387 (43.7%) patients were transferred from other hospitals, and 113 (34.9%) patients were admitted via the OPD. During the pandemic, the proportion of patients transferred from other hospitals decreased (before: 327 patients (45.1%); during: 60 patients (37.1%); *p* < 0.070). In the surgical ICU, 150 (40.3%) of 372 patients were admitted via the ED, 160 (43.0%) patients were transferred from other hospitals, and 62 (16.7%) patients were admitted via the OPD. There was no change in the route of surgical ICU admission during the pandemic (Fig. 3B, Supplemental Table 3).

### Medical ICU

The mean patient age was 67 ± 13.9 years old and 59% were male. Table 1 shows changes in baseline patient characteristics admitted to the ICU before and during the pandemic. There was no change in patient demographics, whereas the proportion of patients with diabetes (before: 44.4%; during: 56.5%; *p* = 0.005) and cancer (before: 19.4%; during: 32.3%; *p* < 0.001) increased during the pandemic. Disease severity upon hospital arrival was not changed, with 50.1% showing circulatory failure, 33.8% showing respiratory failure, and 34.9% with kidney failure. There was no change in disease severity or laboratory results at the time of CRRT initiation in the ICU.

**Table 1:** Comparisons of patient characteristics before and during the Coivd-19 pandemic

	Medical (***N*** = 886)					Surgical (***N*** = 373)				
	No. of data available	Total ***N*** = 886	Before ***N*** = 725	After ***N*** = 161	***P***-value	No. of data available	Total ***N*** = 373	Before ***N*** = 306	After ***N*** = 67	***P***-value
**Demographics**										
Age, year	885	67.0 ± 13.9^*^	66.9 ± 12.1	67.5 ± 12.8	0.604	372	61.2 ± 15.1	60.8 ± 15.2	62.9 ± 14.4	0.303
Male, %	886	521(59.0)^*^	427(58.9)	96(59.6)	0.873	373	274(73.5)	224(73.2)	50(74.6)	0.811
Height, cm	856	163.0 ± 9.4^*^	162.8 ± 9.6	164.1 ± 8.5	0.106	363	165.2 ± 8.9	164.8 ± 8.9	166.5 ± 8.7	0.166
Weight, kg	758	63.2 ± 29.0^*^	63.0 ± 31.3	64.1 ± 12.8	0.499	309	67.8 ± 15.6	67.6 ± 15.5	69.3 ± 16.1	0.478
BMI, kg/m2	750	23.8 ± 12.4	23.8 ± 13.5	23.7 ± 3.8	0.925	305	24.8 ± 4.7	24.7 ± 4.7	25.2 ± 4.8	0.524
**Comorbidities**										
DM, N(%)	881	411(46.7)^*^	320(44.4)	91(56.5)	0.005	363	112(30.9)	90(30.4)	22(32.8)	0.697
HT, N(%)	881	460(52.2)^*^	381(52.9)	79(49.1)	0.377	363	141(38.8)	108(36.5)	33(49.3_	0.053
CVD, N(%)	881	280(31.8)^*^	220(30.6)	60(37.3)	0.098	363	71(19.6)	54(18.2)	17(25.4)	0.184
CKD, N(%)	881	138(15.7)	105(14.6)	33(20.5)	0.062	363	43(11.8)	30(10.1)	13(19.4)	0.034
Liver, N(%)	881	100(11.4)	76(10.6)	24(14.9)	0.116	363	43(11.8)	35(11.8)	8(11.9)	0.979
Lung, N(%)	881	81(9.2)	67(9.3)	14(8.7)	0.807	363	22(6.1)	19(6.4)	3(4.5)	0.548
Cancer, N(%)	884	192(21.7)^*^	140(19.4)	52(32.3)	< 0.001	373	50(13.4)	37(12.1)	13(19.4)	0.112
**Disease severity upon hospital arrival**										
Organ failure										
Circulatory	871	436(50.1)	361(50.8)	75(46.6)	0.329	318	198(62.3)^ǂ^	157(60.6)	41(69.5)	0.204
Respiratory	872	295(33.8)	233(32.8)	62(38.5)	0.165	318	189(59.4)^ǂ^	155(59.8)	34(57.6)	0.754
Kidney failure	886	309(34.9)^*^	262(36.1)	47(29.2)	0.094	373	62(16.6)	48(15.7)	14(20.9)	0.300
**Disease severity at CRRT**										
Hours from ICU to RRT	882	0(0, 1)	0(0, 1)	1(0, 2)	0.295	373	1(0, 4)	1(0, 4.5)	1(0, 3)	0.141
Organ failure										
Circulatory	883	576(65.2)	474(65.7)	102(63.4)	0.580	372	270(72.6)^ǂ^	220(72.1)	50(74.6)	0.678
Respiratory	883	453(51.3)	365(50.6)	88(54.7)	0.346	372	300(80.6)^ǂ^	249(81.6)	51(76.1)	0.300
Number	883	2.2 ± 0.8	2.2 ± 0.8	2.2 ± 0.8	0.813	372	2.5 ± 0.7^ǂ^	2.5 ± 0.7	2.5 ± 0.7	0.748
**Lab values at CRRT initiation**										
WBC, 10E3/uL	883	14.3 ± 11.7	14.4 ± 11.8	13.7 ± 11.5	0.463	370	11.5 ± 8.4	11.5 ± 8.3	11.3 ± 8.9	0.845
Hb, g/dL	882	10.2 ± 3.8	10.3 ± 3.9	10.3 ± 2.5	0.137	370	10.2 ± 4.3	10.2 ± 4.6	10.1 ± 1.8	0.911
PLT, 10E3/uL	883	155.3 ± 110.8	156.4 ± 110.3	150.6 ± 113.0	0.547	371	124.3 ± 79.3^ǂ^	127.2 ± 82.7	112.2 ± 59.8	0.137
TP, g/dL	879	5.9 ± 5.4	5.9 ± 5.9	5.5 ± 1.2	0.280	372	5.0 ± 1.0^ǂ^	5.0 ± 0.9	4.9 ± 0.9	0.494
Albumin, g/dL	880	3.0 ± 0.7	3.0 ± 0.7	3.0 ± 0.7	0.886	372	2.9 ± 0.6	2.9 ± 0.5	2.9 ± 0.8	0.575
BUN, mg/dL	882	62.7 ± 38.7^*^	61.5 ± 38.9	67.9 ± 37.3	0.055	372	48.7 ± 33.1	47.7 ± 32.6	53.1 ± 34.9	0.230
Cr, mg/dL	882	3.8 ± 4.1^*^	3.7 ± 4.3	4.1 ± 3.0	0.259	372	2.9 ± 2.0	2.8 ± 2.1	3.1 ± 1.7	0.262
Na, mmol/L	883	135.4 ± 7.8^*^	135.1 ± 7.8	136.5 ± 8.1	0.049	372	140.3 ± 6.3	140.6 ± 6.1	139.1 ± 6.7	0.072
K, mmol/L	883	4.7 ± 4.4	4.8 ± 4.8	4.6 ± 1.0	0.719	372	4.5 ± 1.0	4.5 ± 1.0	4.6 ± 1.0	0.581
tCO2, mmol/L	754	15.4 ± 7.4^*^	15.4 ± 7.6	15.5 ± 5.4	0.929	224	18.9 ± 11.9	19.1 ± 12.0	16.4 ± 6.7	0.530
PT, INR	752	1.8 ± 1.9	2.1 ± 3.6	1.8 ± 1.3	0.058	323	1.7 ± 1.1	1.7 ± 1.2	1.8 ± 0.6	0.728

*Abbrevations*: *N data available* Number of data available, *BMI* body mass index, *DM* diabetes, *HT* hypertension, *CVD* cardiovascular disease, *CKD* chronic kidney disease, *WBC* white blood cell, *Hb* hemoglobin, *PLT* platelet, *TP* total protein, *BUN* blood urea nitrogen, *Cr* creatinine, *Na* sodium, *K* potassium, *PT* prothrombin time

^*^p < 0.05 compared to surgical patients

^ǂ^*p* < 0.05 compared to medical patients

Table 2 shows changes in the time sequence in the process of care by period. During the pandemic, the median time from hospital arrival to ICU admission was 0 (0, 5) days and the median time from ICU admission to CRRT was 1 (0, 2) days, which were not delayed compared to before the pandemic. A total of 147 out of 161 patients (95.5%) received an adequate dose of CRRT, with a median duration of 3(2, 5) days, as before the pandemic.

**Table 2:** Comparisons of process of care before and during pandemic, in the critically ill patients without Covid-19 infection

	Medical (***N*** = 886)					Surgical (***N*** = 373)				
	N	Total	Before	After	***P***-value	N	Total	Before	After	***P***-value
Days from hospital arrival to ICU, median(IQR)	882	0(0, 2)	0(0, 2)	0(0, 5)	0.693	373	0(0, 4.5)	0(0, 3.6)	0(0, 2)	0.406
Days from ICU to CRRT, median(IQR)	882	0(0, 2)	0(0, 1)	1(0,2)	0.341	373	1(0, 4)	1(0, 4.25)	1(0,3)	0.119
Actually delivered dose > 25 ml/kg/hr	852	818(96.0)	671(96.1)	147(95.5)	0.698	367	358(97.5)	292(97.3)	66(98.5)	0.574
CRRT duration, days	886	3(2, 6)	3(2, 6)	3(2, 5)	0.860	373	6(3, 12)	6(3, 12)	5(3, 10)	0.396
Time from hospital arrival to interventions/operations, median (IQR) hr						334	4(1. 65)	5(1–65.0)	4(1–63.5)	0.614

*Abbreviations*: *ICU* intensive care unit, *CRRT* continuous renal replacement therapy

Patient outcomes during the pandemic were compared in Table 3. During the pandemic, median days of hospital admission was 20 (8.3, 37.5) days, and there was no change in ICU (before: 47.9%; during: 43.4%, *p* = 0.318) or in-hospital mortality rate (before: 52.7%; during: 48.7%, *p* = 0.371). In the univariable and multivariable analyses, the pandemic had no impact on in-hospital mortality in medical ICU patients (Table 4). Cancer (HR: 1.509 (1.212–1.879]), low serum albumin at CRRT (HR: 0.733 [9.628–0.855]), respiratory (HR: 1.277 [1.002–1.627]), and circulatory (HR: 1.504 [1.183–1.912]) failure upon hospital arrival were associated with increased in-hospital mortality (Supplemental Table 4).

**Table 3:** Comparisons of patient outcome before and during the pandemic

	Medical					Surgical				
	N	Total (***N*** = 886)	Before (***N*** = 725)	After (***N*** = 161)	***P***-value	N	Total (***N*** = 373)	Before (***N*** = 306)	After (***N*** = 67)	***P***-value
Admission duration, days, median(IQR)	852	18(7,37)^*^	19(7, 37.8)	20(8.3, 37.5)	0.646	350	27(10, 67)	30(13, 68)	21(7, 43)	0.021
ICU mortality, N(%)	858	404(47.1)	338(47.9)	66(43.4)	0.318	352	184(52.4)	150(51.4)	34(56.7)	0.454
In-hospital mortality, N(%)	858	446(52.0)	373(52.7)	73(48.7)	0.371	352	191(54.3)	156(53.6)	35(57.4)	0.591

^*^p < 0.05 compared to surgical patients

**Table 4:** Uni- and Multi-variable analyses predicting the effects of Covid-19 pandemic on the in-hospital mortality of critically ill patients without Covid-19 infection

	Medical Department		Surgical Department	
	HR	95% CI	HR	95% CI
Univariable	0.991	0.769–1.276	1.478	1.020–2.143
Full Model^a^	0.916	0.704–1.191	1.130	0.740–1.725
Final Model^b^	1.002	0.778–1.292	1.178	0.783–1.772

^a^Full model included Age, Sex, CKD, Cancer, Serum albumin level at CRRT initiation, Circulatory, and Respiratory failure upon hospital arrival

^b^Final model included Age, Cancer, Serum albumin level at CRRT initiation, Circulatory, and Respiratory failure at admission in Medical department. In Surgical department, Age, Sex, and Respiratory failure upon hospital arrival was adjusted

### Surgical ICU

Compared to medical patients, surgical patients were younger (medical: 64.7; surgical: 61.2, *p* < 0.05), had higher proportions of males (medical: 59%; surgical: 73.5%, *p* < 0.05), and had a lower proportion of diabetes (medical: 46.7; surgical: 30.95%, *p* < 0.05), hypertension (medical: 52.2%; surgical: 38.8%, *p* < 0.05), cardiovascular disease (medical: 31.8%; surgical: 19.6%, *p* < 0.05), and cancer (medical: 21.7%; surgical: 13.4%, *p* < 0.05). There were no changes in demographics or comorbidities due to the pandemic, except for the decrease in the proportion of CKD during the pandemic. Circulatory and respiratory failure was more common in surgical patients compared to medical patients at both hospital arrival and CRRT initiation. There was no change in disease severity due to the pandemic in surgical patients (Table 1).

**Fig. 4:**
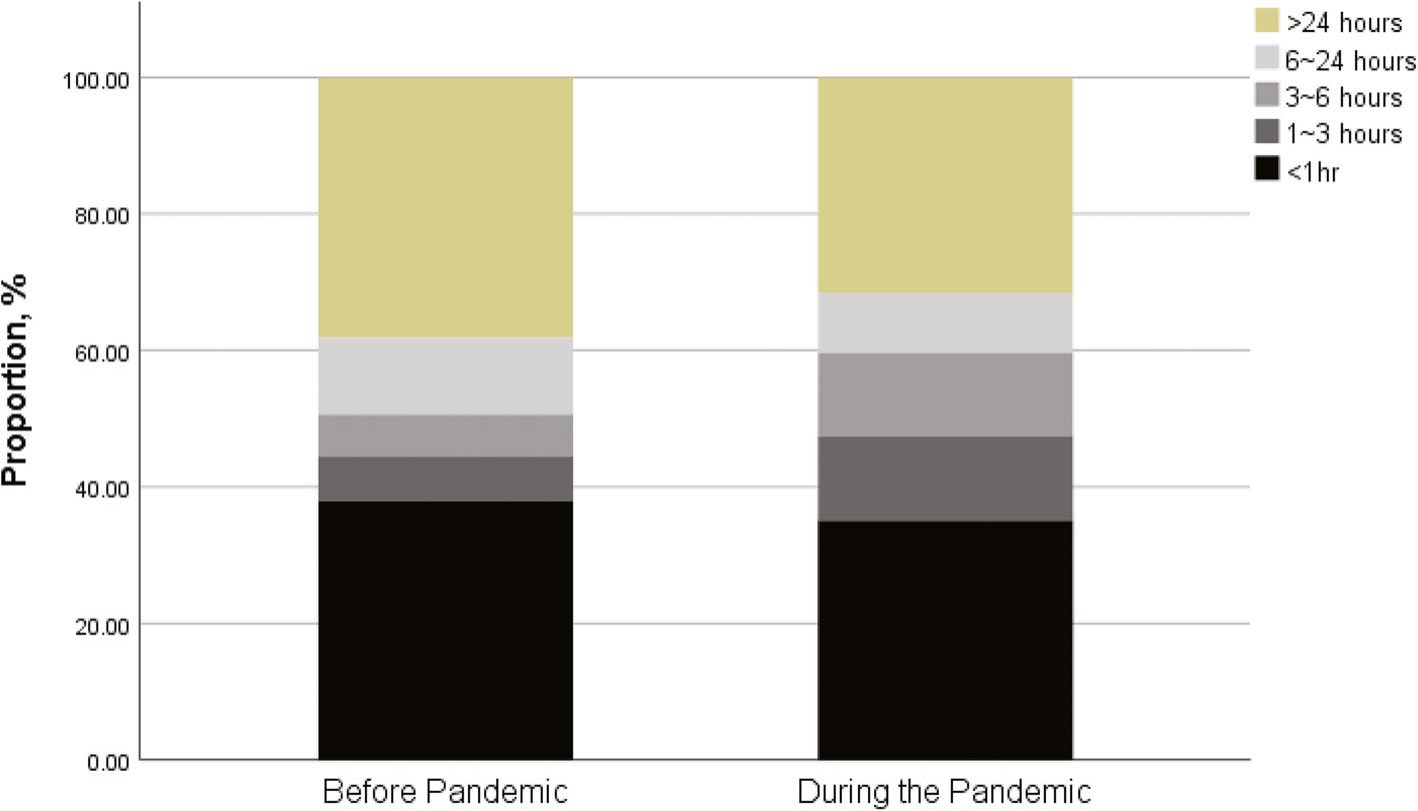
The numbers of interventions/surgeries implemented over time before and during the pandemic. Black: within 1 h of hospital arrival; dark gray: within 1 to 3 h; mid-gray: within 3 to 6 h; light gray: within 6 to 24 h; olive green: after 24 h

There was no delay in the process of care during the pandemic (Table 2). Median time from hospital arrival to ICU was 0 (0, 2 days), and ICU arrival to CRRT initiation was 1 (0, 3) days. A total of 66 out of 67 (98.5%) patients received an adequate dose of CRRT with a median duration of 5 (3, 10) days, which was not different compared with before the pandemic. Information on the time from hospital arrival to intervention/operation was available in 334 (89.5%) out of 373 patients. During the pandemic, the time from hospital arrival to intervention/operation was 4 (1, 63.5) hours, which was similar to before the pandemic (5 (1, 65) hours) (Table 2). When categorized by time, 35.1% of interventions/operations were performed within 1 hour of hospital arrival, 47.4% were performed within 3 hours of hospital arrival, and 59.7% were performed within 6 hours of hospital arrival (Fig. 4) during the pandemic.

Compared to before the pandemic, admission duration was decreased (before: 30 (13, 68) days; during: 21 (7, 43) days, *p* = 0.021) in surgical patients. However, there was no change in the proportion of ICU or in-hospital mortality during the pandemic (Table 3). In the cox-regression univariable analysis, the hazard ratio of the pandemic on in-hospital mortality was 1.478 (1.020, 2.143), whereas in the multivariable analyses adjusted for age, sex, and respiratory failure upon hospital arrival, the pandemic had no effect on mortality (1.178 [0.783, 1.772]) (Table 4). Old age (HR: 1.025 [1.013–1.037]), female sex (HR: 0.620 [0.441–0.873]), and respiratory failure upon hospital arrival (HR: 1.834 [1.298–2.601]) were associated with increased in-hospital mortality in surgical patients (Supplemental Table 4).

## Discussion

During the COVID-19 pandemic, non-COVID 19 hospital visits significantly decreased globally. In Spain [9], 40% fewer patients with ST-elevation myocardial infarction (MI) were observed during the early phase of the pandemic. In the USA [10], the weekly rates of hospitalization for acute MI decreased by up to 48%. In South Korea, a significant decrease in the number of outpatient clinic visits in the spine department was reported in March 2020 compared to those from 2017 to 2019 [11]. Kim et al. [12] reported an increase in the proportion of no-show appointments in the rheumatology department during the early phase of the pandemic. From a nationwide population-based study by Sung et al. [13], the number of ED visits in AMI patients was reduced by approximately 10% compared with that in 2018 and 2019. Explanations for the decrease in hospital visits may be the climate of COVID-19-related fears, the desire to maintain social distancing, and the desire to avoid contact with infected individuals [13, 14].

Reduced hospital access of non-COVID 19 patients, especially critically ill patients, may result in worse patient outcomes and indirectly increase overall mortality during the pandemic. After the early phase of the pandemic, the WHO recommended nation and sub-nation level authorities to implement strategies to maintain essential health care services during the pandemic [15].

Hospital reorganization [16] is one of the solutions for the maintenance of health care services during the pandemic. Institutional-level reorganization model was suggested by Deana et al. [16]. They said that the space, human resources, and technology needed to be diverted from standard departments to those dedicated to COVID-19 patients.

When the situation is under control, the original dispositions can be restored. National-level hospital reorganization can be conducted by the government, by dividing national hospitals into “COVID-19 specific hospitals” which is focusing on the treatment of COVID-19 infected patients, and “non-COVID-19-specific hospitals” which hands all other patients.

The Korean government designated a national safe hospital, where it operates dedicated areas for respiratory disease, screening, and triage in suspected COVID-19 patients to prevent cross infection from respiratory to non-respiratory cases [3]. Our institute was designated as a national-safe hospital from February 25, 2020, and took part in the continuation of essential health care services for non-COVID 19 patients. As a national safe hospital, institutional level challenges in treating critically ill patients during the pandemic involved preventing nosocomial COVID-19 infections and implementing time-sensitive interventions/operations in COVID-19 undetermined patients. As symptom presentation of COVID-19 varies, and some COVID-19 infected patients are asymptomatic [17–19], (only) symptomatic screening for COVID-19 infections misses some infected patients. Moreover, transmission from patient to ICU health care workers can result in fatal outcomes. To prevent nosocomial infections, we performed COVID-19 PCR tests on patients prior to ICU admission or intervention/operation. During the early phase of the pandemic, when COVID-19 positive cases were sporadic, we selectively performed COVID-19 PCR tests based on the presence of respiratory symptoms, fever, and history of contacting COVID-19 confirmed individuals. With the increasing case numbers in our community over time, we tested all patients receiving interventions/operations or being admitted to the ICU. However, PCR tests require 6 h [7], and some patients require immediate care. In these cases, we performed intervention/surgery wearing a level D protection suit [8]. For patients requiring urgent ICU admission, we placed the patient in an isolated room located in the ICU, and then relocated them to the open ICU ward after confirmation of being negative for COVID-19.

Our model is similar one implemented by Piani et al. [20] in Italy. The hospital was divided into green and blue zones. The green zone admitted only COVID-19-negative patients; the blue zone cared for those with suspected or confirmed COVID-19 infections. The cited authors stressed that possible COVID-19-positive patients should be regarded as positive until a negative PCR test is obtained. This applied to all patients (thus those requiring operation or ICU admission). Our strategy mirrors that triage system; personnel performing time-sensitive interventions or surgeries wore Level-D protective equipment.

Our results show that time sequences in the process of care were maintained during the pandemic. The median time from hospital arrival to ICU admission was 0 days, and the median time from ICU admission to CRRT initiation was 1 day. Among the 67 surgical patients, 35.1% of patients received interventions/operations within 1 hour and 59.7% of the patients received interventions/operations within 6 hours, which did not differ from the times before the pandemic. Until April 30, 2021, there was no COVID-19 transmission from patient to ICU or intervention/surgical health care workers, and there were no cases of nosocomial infections from health care workers to patients during the ICU stay.

Some limitations of our study include that we could not evaluate hospital accessibility since we only counted patients admitted to the ICU, and patients who required ICU care but were unable to access the ICU were not counted. Statistically, the annual number of patients admitted to the medical ICU did not change; however, it was expected to increase as one ICU near our institute was closed from the very early phase of the pandemic after being designated a COVID-19 hospital. We suspect ICU accessibility for critically ill, non-COVID 19 infected patients was reduced around our area during the pandemic in both surgical and medical patients. A larger epidemiologic study is required to address this issue.

Second, we did not count ICU patients without CRRT. Since only a limited proportion (6.6%) of ICU admitted patients received CRRT during the course of the disease, the results cannot be interpreted as a reflection of the whole critical care system during the pandemic. Nevertheless, our data are valuable because patients requiring CRRT are the most severely ill group [21–23], having the highest mortality rate among ICU admitted patients [24, 25]. Therefore, successful maintenance of critical care in this group is directly associated with reducing non-COVID-19 associated mortality during the pandemic.

## Conclusions

In conclusion, during the pandemic, the critical care system for non-COVID 19, critically ill patients requiring CRRT was well maintained in our institute. Further nationwide studies testing the continuity of the maintenance of essential health care systems are required.

## Supplementary Information

**Additional file 1: Supplemental Table 1.** Intensive care units of our institute. **Supplemental Table 2.** Annual number of ICU admissions and CRRT procedures performed between January 1 2016 and December 31 2020 (A) and changes in these parameters during the pandemic (B). **Supplemental Table 3.** Changes in the routes of admission during the pandemic. **Supplemental Table 4.** Factors associated with in-hospital mortality of critically ill patients, stratified by the department of care.

## Data Availability

The datasets generated and/or analyzed during the current study are not publicly available due to the upcoming analyses concerning other study setups but are available from the corresponding author (rheeharin@pusan.ac.kr) upon reasonable request.

## Abbreviations

WHO: World Health Organization
ICU: Intensive care unit
CRRT: Continuous renal replacement therapy
PPE: Personal protection equipment
CT: Computed tomography
ED: Emergency department
OPD: Outpatient department
MI: Myocardial infarction

## References

[1] Worldometer (2021). Covid-19 Coronavirus pandemic, Weekly Trends.

[2] Worldometer (2021). Covid-19 coronavirus pandemic, Countries, South Korea.

[3] Kang J, Jang YY, Kim J, Han SH, Lee KR, Kim M, Eom JS (2020). South Korea's responses to stop the COVID-19 pandemic. Am J Infect Control.

[4] Kang HKS, Kim E (2020). COVID-19 health system response monitor: Republic of Korea.

[5] Khetrapal Singh P, Ofrin RH (2020). Quo vadis after COVID-19: a new path for global emergency preparedness?. WHO South East Asia J Public Health.

[6] Elston JW, Cartwright C, Ndumbi P, Wright J (2017). The health impact of the 2014-15 Ebola outbreak. Public Health.

[7] PowerChek™ 2019-nCoV Real-time PCR Kit. https://www.fda.gov/media/140069/download. Accessed May 2021.

[8] Personal Protective Equipment; United States Environmental Protection Agency. https://www.epa.gov/emergency-response/personal-protective-equipment. Accessed May 2021.

[9] De Rosa S, Spaccarotella C, Basso C, Calabro MP, Curcio A, Filardi PP, Mancone M, Mercuro G, Muscoli S, Nodari S (2020). Reduction of hospitalizations for myocardial infarction in Italy in the COVID-19 era. Eur Heart J.

[10] Solomon MD, McNulty EJ, Rana JS, Leong TK, Lee C, Sung SH, Ambrosy AP, Sidney S, Go AS (2020). The Covid-19 pandemic and the incidence of acute myocardial infarction. N Engl J Med.

[11] Ham CH, Moon HJ, Kim JH, Park YK, Lee TH, Kwon WK (2020). Coronavirus disease (COVID-19) outbreak and its impact on spinal daily practice : preliminary report from a single (Regional) University Hospital in Republic of Korea. J Korean Neurosurg Soc.

[12] Kim Y, Ahn E, Lee S, Lim DH, Kim A, Lee SG, So MW (2020). Changing patterns of medical visits and factors associated with no-show in patients with rheumatoid arthritis during COVID-19 pandemic. J Korean Med Sci.

[13] Sung HK, Paik JH, Lee YJ, Kang S (2021). Impact of the COVID-19 outbreak on emergency care utilization in patients with acute myocardial infarction: a Nationwide population-based study. J Korean Med Sci.

[14] Boserup B, McKenney M, Elkbuli A (2020). The impact of the COVID-19 pandemic on emergency department visits and patient safety in the United States. Am J Emerg Med.

[15] Maintaining essential health services: operational guidance for the COVID-19 context. Geneva: World Health Organization 2020. https://www.who.int/publications/i/item/WHO-2019-nCoV-essential-health-services-2020.1.

[16] Deana C, Rovida S, Orso D, Bove T, Bassi F, De Monte A, Vetrugno L (2021). Learning from the Italian experience during COVID-19 pandemic waves: be prepared and mind some crucial aspects. Acta Biomed.

[17] Liu YC, Liao CH, Chang CF, Chou CC, Lin YR (2020). A locally transmitted case of SARS-CoV-2 infection in Taiwan. N Engl J Med.

[18] Kimball A, Hatfield KM, Arons M, James A, Taylor J, Spicer K, Bardossy AC, Oakley LP, Tanwar S, Chisty Z (2020). Asymptomatic and Presymptomatic SARS-CoV-2 infections in residents of a long-term care skilled nursing facility - King County, Washington, march 2020. MMWR Morb Mortal Wkly Rep.

[19] Chan JF, Yuan S, Kok KH, To KK, Chu H, Yang J, Xing F, Liu J, Yip CC, Poon RW (2020). A familial cluster of pneumonia associated with the 2019 novel coronavirus indicating person-to-person transmission: a study of a family cluster. Lancet.

[20] Tommaso Piani DZ, Deana C (2021). Sliding doors during COVID-19: choose the right one!. J Emerg Med Trauma Acute Care.

[21] Boussekey N, Chiche A, Faure K (2012). Section 5: Dialysis interventions for treatment of AKI. Kidney Int Suppl.

[22] Mehta RL (2005). Continuous renal replacement therapy in the critically ill patient. Kidney Int.

[23] See E, Ronco C, Bellomo R. The future of continuous renal replacement therapy. Semin Dial. 2021;34(6):576-585. 10.1111/sdi. 10.1111/sdi.12961 33609407

[24] Prowle JR, Bellomo R (2010). Continuous renal replacement therapy: recent advances and future research. Nat Rev Nephrol.

[25] Metnitz PG, Krenn CG, Steltzer H, Lang T, Ploder J, Lenz K, Le Gall JR, Druml W (2002). Effect of acute renal failure requiring renal replacement therapy on outcome in critically ill patients. Crit Care Med.

