# Supplementary material for: Maintenance of the critical care system during the pandemic in non-COVID-19 patients requiring continuous renal replacement therapy: a single center experience
Source: BMC Emerg Med. 2022 Aug 1;22:138. doi: 10.1186/s12873-022-00693-7 (PMC9342846; doi:10.1186/s12873-022-00693-7)
Supplement: Supplementary file 1 — Additional file 1: Supplemental Table 1. Intensive care units of our institute. Supplemental Table 2. Annual number of ICU admissions and CRRT procedures performed between January 1 2016 and December 31 2020 (A) and changes in these parameters during the pandemic (B). Supplemental Table 3. Changes in the routes of admission during the pandemic. Supplemental Table 4. Factors associated with in-hospital mortality of critically ill patients, stratified by the department of care. [file 12873_2022_693_MOESM1_ESM.docx]

**Supplement Table 1. Intensive care units of our institute**

|  | **Type of ICU** | **No. of beds** | **Type of patients** |
| --- | --- | --- | --- |
| **Surgical ICU** | **NSICU** | 13 | Patients requiring any kind of neurosurgery |
|  | **Trauma-ICU1** | 14 | Any patient with critical trauma |
|  | **Trauma-ICU2** | 14 | Any patient with critical trauma |
|  | **Trauma-ICU3** | 14 | Any patient with critical trauma |
|  | **SICU** | 10 | Patients requiring any kind of surgery |
| **Medical ICU** | **CCU** | 14 | Patients with cardiovascular disease |
|  | **EICU** | 14 | Patients with any intoxication or any kind of medical disease |
|  | **RICU** | 12 | Patients with respiratory care or any kind of medical disease |

Abbreviations: ICU, intensive care unit; NSICU, neurosurgical ICU; SICU, surgical ICU; CCU, coronary care unit; EICU, emergent ICU; RICU, respiratory ICU

**Supplement Table 2. Annual number of ICU admitted and CRRT received patients between Jan.1, 2016 and Dec.31, 2020 (A) and changes in number during the pandemic (B)**

| **(A)** | **Medical** | | | **Surgical** | | |
| --- | --- | --- | --- | --- | --- | --- |
|  | **No. patient**  **ICU admitted** | **No. patient**  **CRRT received*** | **% CRRT among ICU admitted** | **No. patient**  **ICU admitted** | **No. patient**  **CRRT received*** | **% CRRT among ICU admitted** |
| **2016** | 1918 | 179 | 9.3 | 2702 | 80 | 2.9 |
| **2017** | 1886 | 182 | 9.6 | 2781 | 78 | 2.8 |
| **2018** | 2401 | 213 | 8.9 | 2755 | 73 | 2.7 |
| **2019** | 2497 | 151 | 6.1 | 2844 | 75 | 2.6 |
| **2020** | 2381 | 134 | 5.6 | 2779 | 56 | 2.0 |
| **Total** | 11,083 | 179 | 9.4 | 13,861 | 362 | 2.6 |

| **(B)** | **Medical** | | | **Surgical** | | |
| --- | --- | --- | --- | --- | --- | --- |
|  | **2016-2019** | **2020** | **P-value** | **2016-2019** | **2020** | **P-value** |
| **No. patient ICU admitted** | 2175.5±318.5 | 2381 | 0.604 | 2770.5±59.1 | 2779.0 | 0.906 |
| **No. patient CRRT received*** | 181.3±25.4 | 134 | 0.194 | 76.5±3.1 | 56 | 0.010 |
| **% CRRT received***  **among ICU admitted** | 8.5±1.6 | 5.6 | 0.220 | 2.7±0.2 | 2.0 | 0.022 |

Foot note: A total of 970 patient admitted to the ICU including 38 CRRT received patient between Jan.2021~Mar.2021 were excluded in this table. *Patients with end-stage renal disease on maintenance dialysis, and patients admitted to the emergency department were excluded.

**Supplement Table 3. Changes in the route of admission during the pandemic**

|  | **Medical (N=886)** | | | | | **Surgical (N=373)** | | | | |
| --- | --- | --- | --- | --- | --- | --- | --- | --- | --- | --- |
|  | **N data**  **available** | **Total**  **N=886** | **Before**  **N=725** | **After**  **N=161** | **P-value** | **N data**  **available** | **Total**  **N=373** | **Before**  **N=306** | **After**  **N=67** | **P-value** |
| ED | 886 | 386(43.6) | 309(42.6) | 77(47.8) | 0.228 | 372 | 150(40.3) | 122(40.0) | 28(41.8) | 0.787 |
| Transferred | 886 | 387(43.7) | 327(45.1) | 60(37.1) | 0.070 | 372 | 160(43.0) | 129(42.3) | 31(46.3) | 0.552 |
| OPD | 886 | 113(12.8) | 89(12.3) | 24(14.9) | 0.365 | 372 | 62(16.7) | 54(17.7) | 8(11.9) | 0.252 |

Abbreviations: ED emergency department, OPD, outpatient clinic

**Supplement Table 4. Factors associated with in-hospital mortality in critically ill patients, stratified by the department**

|  | **HR(95% CI)** | **p-value** |
| --- | --- | --- |
| **Medical^a^** |  |  |
| Cancer | 1.509(1.212, 1.879) | <0.001 |
| Serum albumin, at CRRT initiation | 0.733 (0.628, 0.855) | <0.001 |
| Required ventilator care upon hospital arrival | 1.277(1.002, 1.627) | 0.048 |
| Required vasopressor upon hospital arrival | 1.504(1.183, 1.912) | 0.001 |
| **Surgical^b^** |  |  |
| Age | 1.025(1.013, 1.037) | <0.001 |
| Male | 0.620(0.441, 0.873) | 0.006 |
| Required ventilator care upon hospital arrival | 1.834(1.013, 1.047) | 0.001 |

^a^Adjusted factors included age, cancer, serum albumin level at CRRT initiation, ventilator requirement at admission, and vasopressor requirement at admission

^b^Adjusted factors included age, sex, and ventilator requirement at admission
